# Micronized Bone Marrow and Niche Preservation: Biological Rationale, Preclinical Evidence, Translational Potential and Limitations

**DOI:** 10.3390/cells15211923

**Published:** 2026-10-22

**Authors:** Edward Jeffrey Donner, Johnny Huard

**Affiliations:** 1 BMAX Medical LLC, Johnstown, CO 80534, USA; 2 Colorado Spine Institute, Johnstown, CO 80534, USA; 3 Elite Regenerative Stem Cell Specialists, Johnstown, CO 80534, USA; 4 The Steadman Philippon Research Institute, Vail, CO 81657, USA

**Keywords:** knee osteoarthritis, bone marrow aspirate concentrate, micronized bone marrow, mesenchymal stem cells, pericytes, bone marrow niche, orthobiologics, extracellular matrix, matricellular proteins, erythrocyte depletion, leukocyte depletion, post-traumatic osteoarthritis

## Abstract

Bone marrow aspirate concentrate (BMAC) has produced inconsistent results in osteoarthritis (OA), with several randomized clinical trials failing to show superiority over saline, hyaluronic acid, or corticosteroid injection. In this Perspective, we propose that these outcomes may reflect, in part, how marrow is harvested and processed rather than an absence of regenerative potential. Aspiration and centrifugation yield a product rich in leukocytes and platelets but poor in mesenchymal stromal cells (MSCs), and do not include marrow’s spatially organized parenchyma—extracellular matrix (ECM), matricellular proteins with their associated extracellular vesicles, growth factors, and cytokines—that specifies cell behavior. We describe BMAX, a niche-preserving, micronized bone marrow parenchyma/stroma product mechanically released from cancellous bone and designed to retain multiple MSC subpopulations, pericytes, hematopoietic progenitors, and megakaryocytes within their native matrix. In a single-donor study in a rat model of post-traumatic knee OA, BMAX significantly reduced pain behavior, improved cartilage regeneration and OARSI scores relative to both vehicle and BMAC, with better regeneration of proteoglycan and type-II collagen staining. Applications beyond OA—including tendon, ligament, intervertebral disk, bone healing, and inflammation-related conditions—are proposed as research hypotheses. The evidence to date is preclinical and hypothesis-generating; it cannot yet separate niche preservation from other differences between the products, and controlled clinical trials are required. If confirmed, the path forward in the treatment of OA, and regenerative medicine in general, may lie less in culture-expanded cell populations or engineering a new cell than in preserving the regenerative unit evolution has already assembled.

## 1. The Unmet Need in Knee Osteoarthritis and the Limits of Current Orthobiologics

Osteoarthritis (OA) can affect almost every joint in the body and will affect the majority of people at some point in their lives. OA is the most prevalent form of arthritis, affecting an estimated 33.2 million US adults, with the knee being the most disabling site, and more than half of individuals with symptomatic knee OA are younger than 65 [1]. The burden is global and escalating: an estimated 595 million people worldwide were living with osteoarthritis in 2020—about 7.6% of the world’s population and a 132% increase since 1990—and knee OA alone affects on the order of 375 million people, a total projected to rise by roughly three-quarters by mid-century as populations age and obesity spreads [2,3]. Available medical treatments—anti-inflammatory medications, activity modification, nutritional supplements, weight loss, and intra-articular injections—can palliate pain, swelling, and disability, but none, including corticosteroid and hyaluronic acid injections, are disease-modifying [4]; repeated corticosteroid use in particular provides only short-term relief and may accelerate cartilage loss [5]. There is no approved disease-modifying OA drug (DMOAD), and care remains symptomatic until the joint is replaced.

Total knee arthroplasty (TKA) is among the safest and most reliable procedures in medicine, and a modern implant can last 20 years or more; approximately 800,000 TKAs are performed annually in the United States, a figure projected to climb steeply as the population ages and obesity-driven, earlier-onset disease increases [6]. Nor is joint replacement a uniquely American phenomenon: primary knee-arthroplasty rates across high-income countries commonly run on the order of 130–230 procedures per 100,000 population per year, national registries in the United Kingdom and Australia have each logged well over a million knee replacements, and international volumes are likewise projected to more than double by 2050 [6]. The aggregate economic burden of OA reaches tens of billions of dollars annually in the United States [6,7]. Arthroplasty is a definitive yet destructive endpoint that does not serve the large population of younger, earlier-stage patients and for which approximately one in five recipients remain dissatisfied with their outcome [8], and roughly 18% of primary TKAs require revision within 25 years [9]. A substantial treatment gap therefore persists—for a durable, joint-preserving, disease-modifying therapy that also avoids the cumulative harms of NSAIDs and repeated corticosteroid injection.

Regenerative medicine has been widely promoted to fill this gap, frequently ahead of controlled data. “Stem cell” clinics, marketing, and social-media promotion have generated considerable hype around cell-based injectables, yet the best controlled evidence shows only modest symptomatic relief at 1 year (mean VAS improvement ≈ 20/100) without convincing evidence of true disease modification—in particular, little regeneration of high-quality articular cartilage. International societies have emphasized that clinical-grade regenerative treatments must be built on a disciplined scientific progression—preclinical in vitro work, then animal studies, then clinical trials—and judged not only on pain and function but on disease-modifying, cartilage-protective or preferably regenerative effect; the European ESSKA-ORBIT effort in particular has stressed that disease modification must be established in rigorous preclinical models before clinical claims are made [10]. Recent high-quality syntheses reinforce this caution: a systematic review and meta-analysis of randomized trials published in *Osteoarthritis and Cartilage* concluded that intra-articular MSCs for chronic knee OA pain “probably provide little to no improvement in pain or physical function” [11]; a contemporaneous editorial in the same journal called for “a reality check,” arguing that for older patients with knee OA the case for autologous stem cells is effectively closed regardless of what is delivered [12]; and a 2025 Cochrane review found that stem-cell injections may slightly improve pain and function versus placebo but without evidence of genuine tissue regeneration [13]. A focused review of bone marrow aspirate concentrate in osteoarthritis reached the same conclusion—across sixteen trials and cohort studies, intra-articular BMAC did not significantly improve pain or function over conventional therapies such as hyaluronic acid or corticosteroid, and structural cartilage benefit was seldom demonstrated—while identifying the heterogeneity and lack of standardization of BMAC preparation as central unresolved problems [14,15]. Additionally, Shapiro et al. demonstrated that intra-articular BMAC and contralateral saline produced equivalent benefit in a prospective, single-blind, placebo-controlled trial where 25 patients with bilateral knee pain from bilateral osteoarthritis were randomized to receive BMAC into one knee and saline placebo into the other [16]. This is consistent with meta-analyses attributing much of the apparent effect of intra-articular injection to contextual and placebo responses [17].

However, and most prominently, in the phase-3 MILES trial (480 participants) autologous BMAC, autologous adipose-derived stromal vascular fraction, and allogeneic umbilical-cord MSCs each produced clinically meaningful pain relief that exceeded the minimal clinically important difference (MCID) and was sustained at one year—evidence that marrow- and adipose-based biologics carry genuine analgesic activity—yet none proved superior to a simple corticosteroid injection, and none improved the MRI structural score [18]. Prespecified analyses further revealed significant treatment-by-age and treatment-by-sex interactions for pain, with BMAC numerically the most effective arm, though MILES deferred the direction of these responder effects to subsequent analysis [18].

These results are frequently interpreted as evidence that cell-based injections are ineffective. We propose a different perspective. The variable that most of these trials did not control is the quality of the biologic itself—how the marrow is harvested, concentrated, and processed. Demonstrating BMAC reaches clinical meaningfulness is itself encouraging in that it shows marrow biologics carry real clinical benefit to build upon. Consistent with this, a recent randomized study found that autologous cell injections were more responsive than both allogeneic cellular products and corticosteroids for knee OA, in a sex- and age-dependent manner, the benefit being greatest in men—particularly those younger than 60—among whom autologous BMAC produced the highest responder rate of any arm, exceeding adipose-derived stromal vascular fraction (SVF) and allogeneic umbilical-cord cells [19]—directly contradicting the notion that all cell-based injectables are equivalent to steroid and, taken together with the significant age- and sex-based treatment interactions in MILES itself [18], implicating the importance of patient selection and, plausibly, biologic source and processing.

The autologous products most commonly offered as alternatives to marrow-based preparations—platelet-rich plasma (PRP), micro-fragmented (micronized) adipose tissue, and adipose-derived stromal vascular fraction (SVF)—each carry their own source- and processing-dependent limitations. PRP, for instance, is typically administered as a course of repeated injections yet delivers only transient, symptomatic benefit: in the randomized, placebo-controlled RESTORE trial it was no better than saline for either knee pain or medial tibial cartilage volume on MRI at one year [20], and even repeated dosing leaves synovial inflammatory and growth-factor profiles unchanged [21]—the signature of a symptomatic rather than a disease-modifying agent. Attempts to improve upon PRP continue, including building systems which lead to higher platelet concentrations as well as supplementation with HA and concentrated A2M, IRAP and other filterable peripheral plasma proteins (conference presentation) [22]. Adipose-derived therapies are compromised since donor obesity degrades the phenotype and therapeutic function of adipose-derived MSCs [23]. Obesity already affects roughly 40% of US adults and is projected to reach nearly one in two by 2030 [24]; the trend is worldwide, with more than one billion people living with obesity globally in 2022—a prevalence that has roughly doubled in adults since 1990 [25]. A large and progressively growing share of the very patients seeking these injections are therefore compromised autologous donors, potentially compromising adipose-derived autologous biologics for an expanding proportion of the population. Direct clinical evidence ties this cell-quality question to outcome: among patients receiving autologous adipose-derived MSCs with high tibial osteotomy for knee OA, although VAS pain did not improve significantly overall (a significant WOMAC reduction emerged only by 24 months), those with the best clinical and structural responses had cells with greater proliferative and colony-forming capacity and fewer senescent cells, so that higher stemness together with lower senescence predicted superior WOMAC and cartilage-repair outcomes at two years [26]. In other words, the disappointing aggregate literature may reflect underpowered trials of poorly optimized and heterogeneous products rather than a failure of the regenerative premise. Other explanations are equally plausible and not mutually exclusive: disease heterogeneity and OA severity, patient selection, variability in dosing and preparation, and differences in rehabilitation protocols may each contribute to disappointing outcomes independently of how the product is processed (Section 7).

J.H.’s own randomized, double-blind, placebo-controlled phase 1/2 trial (ClinicalTrials.gov NCT04815902; 120 patients with Kellgren–Lawrence grade 2–4 knee OA) speaks directly to the question of what to optimize: using a 2 × 2 factorial design it tested whether adding the senolytic fisetin and the antifibrotic losartan could enhance a single intra-articular BMAC injection, and found that neither agent—alone or in combination—improved pain, patient-reported outcomes, or MRI T2 cartilage measures over BMAC alone, even as symptoms improved across all arms through 18 months (reported to date in abstract form) [27]. These results are consistent with the premise of the program described here—that the more important variable may be the quality and composition of the biologic itself rather than pharmacologic adjuncts.

Notably, the culture-expanded umbilical-cord MSC arm was numerically among the least-effective arms in the MILES study, consistent with the fact that culture expansion drives a progressive accumulation of senescent cells in both adipose-derived and marrow-derived MSCs, further degrading their secretome and regenerative capacity, and an increasing number of findings support the hypothesis that MSCs undergo a shift from a pro-regenerative to a chronic inflammatory phenotype and secretome throughout in vitro expansion. Senolytic agent fisetin was found to attenuate cellular senescence accumulation during culture expansion of human adipose-derived stem cells [28,29]. The limitations of culture-expanded MSCs are further underscored by the fact that, despite decades of research and development, only a limited number of products have been approved worldwide—including just one in the US, Ryoncil (remestemcel-L), which the FDA approved in December 2024 as the first mesenchymal stromal cell therapy, for the narrow indication of steroid-refractory acute graft-versus-host disease in pediatric patients [30].

Additionally, allogeneic culture-expanded MSCs have been mischaracterized as “immune privileged,” creating another reason allogeneic off-the-shelf products, including birth-tissue-derived orthobiologics marketed as “young” stem-cell products, have frequently not met expectations in controlled trials. Li et al. contrast autologous MSCs, which reduce the risk of rejection but vary in cell number and proliferative capacity, with allogeneic MSCs (allo-MSCs), which “possess broad clinical applications unconstrained by donor physiology. Nonetheless, preclinical and clinical investigations highlight that transplanted allo-MSCs are subject to immune attack from recipients. These cells exhibit antiinflammatory and proinflammatory phenotypes contingent on the microenvironment. Notably, the proinflammatory phenotype features enhanced immunogenicity and diminished immunosuppression, potentially triggering allogeneic immune reactions that impede long-term clinical efficacy” [31].

Induced pluripotent stem cells (iPSCs) are likewise promoted as a next-generation source, yet despite nearly two decades of development since their discovery no iPSC-derived therapy has achieved routine regulatory approval; clinical use remains confined to trials and, in Japan, a limited number of conditional approvals [32]. The recent SCUlpTOR trial of an iPSC-derived MSC product (Cynata CYP-004) for medial tibiofemoral knee OA, which the author of the Osteoarthritis and Cartilage editorial highlighted as a hopeful next-generation therapy [12], illustrates the challenge: according to the product developer’s announcement of top-line results (June 2026; peer-reviewed publication pending), it missed both of its 24-month co-primary endpoints—pain and cartilage thickness—with no evidence of reduced cartilage loss [33].

The primary objective of this Perspective is to examine a single, testable hypothesis: that the disappointing results of marrow-derived orthobiologics in knee OA reflect not an intrinsic failure of MSCs, but the loss during conventional harvest and processing of the native bone-marrow niche—its matricellular signaling proteins, extracellular matrix, perivascular and parenchymal populations, and matrix-bound anti-inflammatory and growth factors—and that a processing method preserving this niche can yield a more biologically active product and fill the treatment gap. We present BMAX, a micronized bone-marrow preparation, as one test of this hypothesis. Throughout, we distinguish between four levels of evidence: established niche biology, mechanistic plausibility, BMAX-specific experimental findings, and demonstrated therapeutic efficacy—the last of which remains, at present, preclinical.

This article is a narrative Perspective rather than a systematic review. Consistent with that format, it does not undertake a formal critical appraisal of each cited study, and the mechanisms of action proposed for BMAX—particularly in Section 2, Section 4.5 and Section 6—are hypotheses derived from established marrow biology rather than mechanisms demonstrated in the product. We have sought to identify the type of evidence behind key statements (clinical trial, systematic review, animal study, or in vitro work) and to label mechanistic arguments as hypothesis-generating where they have not been tested in BMAX itself. Our objective is to stimulate further research and interest in this understudied approach. The literature was identified through searches of PubMed and from the authors’ knowledge of the field, prioritizing controlled clinical trials, systematic reviews, and mechanistic studies published through 2026; we have sought to include null and contradictory findings—including negative trials of BMAC, PRP, SVF, and allogeneic and iPSC-derived products—alongside supportive mechanistic and preclinical evidence. No formal systematic-review protocol, predefined search string, or quantitative synthesis was applied, so citation selection reflects editorial judgment; this is acknowledged among the limitations in Section 7.

## 2. What Conventional Harvest and Processing Miss: The Marrow Matrix as a Functional Signaling Scaffold

Bone marrow aspirate concentrate is produced by aspirating liquid marrow and centrifuging it to a buffy-coat concentrate. This approach is simple and minimally manipulated, but it also omits biologic material important to the product’s therapeutic value. Aspiration preferentially samples the sinusoidal blood compartment and dilutes the specimen with peripheral blood, yielding a red-cell-rich concentrate in which the therapeutically relevant MSCs are rare. Although Hernigou has described more effective methods to obtain increased numbers of MSCs [34], aspiration cannot efficiently recover the spatially organized, matrix-embedded parenchymal and stromal populations that reside within trabecular bone. High-resolution single-cell mapping of human marrow has shown that its MSC compartment is far more diverse than previously appreciated, with the most proliferative, stem-like subsets localized near trabecular and endosteal surfaces, and the authors concluded explicitly that “there is previously unrepresented and functionally consequential diversity within human bone marrow MSCs that cannot be captured efficiently from aspirate samples” [35]. A harvest confined to aspirate therefore systematically omits some of the most regeneratively competent cells, and centrifugation further separates whatever might be recovered from its native ECM and accessory populations.

What is missed by aspiration is best described not as ECM in isolation but as the marrow parenchyma and its niche constituents—the matrix scaffold together with its matricellular proteins, bound cytokines and chemokines, accessory and progenitor cells, and the physical framework that supports cell–cell signaling, including the nanotube- and vesicle-mediated exchange discussed in Section 6.

The matrix biology summarized below is well established in native marrow; whether these functions persist after micronization and intra-articular delivery has not yet been tested. The stroma with its ECM is an information-rich signaling scaffold. High-resolution mapping of marrow shows that its non-hematopoietic compartment is a spatially organized mesh of extracellular matrix, adhesion molecules, and matrix-bound factors that actively conditions stromal-cell behavior [36,37]. Its glycosaminoglycans (GAGs)—heparan sulfate, chondroitin/dermatan sulfate, and hyaluronan, carried on proteoglycan core proteins such as perlecan, the syndecans, and glypicans—are the functional unit of this scaffold. Heparan-sulfate proteoglycans bind, protect, and present growth factors to their receptors: heparan sulfate is an obligate co-receptor for FGF-2, forming the FGF–FGFR–heparan-sulfate ternary complex required for receptor dimerization and signaling [38,39], and it likewise co-regulates BMP, VEGF, PDGF, and Wnt; perlecan is specifically required for FGF-2 signaling within the stem-cell niche [40]. Crucially, it is the pattern of sulfation, not the bulk quantity of GAG, that dictates which factors are bound and presented—small changes in heparan-sulfate sequence switch ligand specificity, for example between FGF-1 and FGF-2 [41,42]. The small leucine-rich proteoglycans decorin and biglycan add a further layer, sequestering TGF-β in the matrix and thereby governing the fate of bone-marrow stromal cells; in their absence, TGF-β signaling is dysregulated and osteoprogenitor output falls [43].

The scaffold instructs cells physically as well as chemically, and the same GAG–proteoglycan network is largely what gives the matrix its mechanical character. The dense fixed negative charge carried by the GAG chains draws in water and generates an osmotic swelling pressure that lets the tissue resist compression and behave as a hydrated, viscoelastic solid rather than a fluid [44]. Cells actively read these properties: tissue cells sense and respond to the stiffness of their substrate [45], matrix elasticity alone is sufficient to direct MSC lineage commitment [46], and the stress-relaxing, viscoelastic behavior of the matrix exerts further control over stem-cell fate and activity [47]. This mechanical information is converted into gene expression through integrin-based adhesions and the actin cytoskeleton, acting on the mechanosensitive transcriptional regulators YAP and TAZ [48]—the same integrin adhesions whose loss triggers the anoikis noted below.

To summarize, delivering cells as a fluid suspension removes the very substrate whose stiffness and viscoelasticity specify their behavior, and expanding them on tissue-culture plastic—on the order of a million-fold stiffer than native marrow—imposes an aberrant mechanical signal that accelerates replicative senescence and erodes potency, whereas soft, tissue-like substrates delay senescence and preserve proliferative and differentiation capacity during expansion [49]. The mechanical environment of the niche is therefore part of the instructive signal, not inert packaging, and it is degraded by precisely the two manipulations—disaggregation into suspension and expansion on plastic—on which conventional and culture-based products depend.

The matrix is also what keeps the therapeutic cells alive and in place. Marrow stromal cells are anchorage-dependent: loss of matrix attachment triggers anoikis, a form of detachment-induced apoptosis [50], and engagement of the fibronectin receptor integrin α5β1 directly suppresses anoikis in bone-marrow MSCs [51]. A biologic that delivers cells as a fluid suspension stripped of their matrix therefore removes the very survival and adhesion cues on which they depend—one mechanistic reason such preparations show poor persistence after injection [52]. Hyaluronan reinforces the point from the niche side: hyaluronan–CD44 signaling anchors hematopoietic and stromal stem cells in the marrow and helps maintain their quiescence and retention [53]; and because hyaluronan is anti-inflammatory at high molecular weight but pro-inflammatory as low-molecular-weight fragments that act through TLR2/TLR4 [54], preserving native high-molecular-weight hyaluronan is an added design consideration for any matrix-retaining product.

Seen this way, the deficiency of conventional preparations is not merely a shortfall of cells but the loss of the scaffold that makes those cells work—their GAG reservoir, their sulfation-encoded factor presentation, and the anchorage that sustains survival and retention. If the preferred operative therapeutic unit is the intact, matrix-embedded, perivascular stromal assembly rather than an isolated cell [55,56], then conventional BMAC delivers a depleted and disaggregated version of the very material it is meant to provide—an interpretation consistent with, though not established by, its underwhelming clinical performance.

## 3. BMAX: A Niche-Preserving Micronized Bone Marrow Orthobiologic

**Figure 1:**
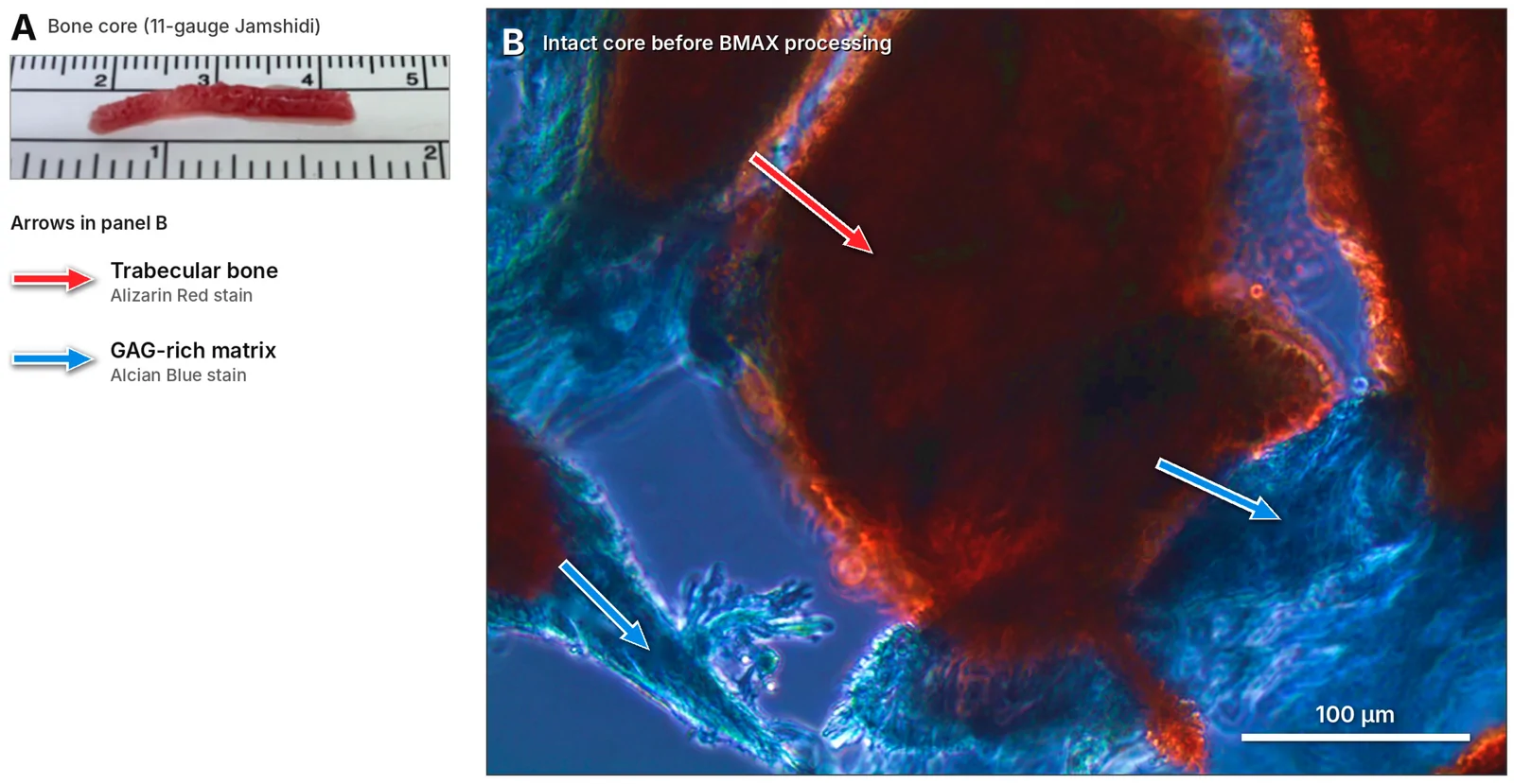
(**A**). Photograph of bone core obtained with an 11GA Jamshidi. (**B**). Micrograph of intact core prior to BMAX™ processing illustrating the trabecular bone stained red with Alizarin Red (red arrow) and attached GAG-rich matrix within sections of trabecular cavities stained blue with Alcian Blue (blue arrows).

BMAX is an autologous orthobiologic produced by mechanically micronizing bone marrow harvested, typically from the ilium, using a Jamshidi needle by removing the trocar prior to entry into the cancellous bone to obtain a core (Figure 1) which includes trabecular bone and its embedded stroma, using a non-enzymatic, point-of-care process that fragments the tissue while preserving the native cell–matrix relationships rather than dissociating them. The design intent is to retain what aspiration and centrifugation miss: the perivascular and endosteal parenchymal and stromal populations, the tissue-specific ECM through which their signals travel, and the accessory cells and platelets that modulate the regenerative response. Retaining that native scaffold is itself functionally consequential: the extracellular matrix to which marrow parenchymal and stromal cells are natively anchored supports their adhesion, survival, proliferation, and retention at the delivery site and helps maintain their stemness—anchorage- and niche-dependent cues that enzymatically dissociated cells stripped into suspension lose—and, consistent with this, BMAX’s preserved-stroma processing yields MSCs with improved adherence and proliferation ex vivo. Because the process is mechanical and non-enzymatic, it avoids the niche disruption caused by enzymatic dissociation and the phenotypic drift and senescence caused by culture expansion, an approach intended to remain consistent with minimal manipulation, although regulatory classification depends on multiple factors beyond processing method and rests with the relevant agencies. Independent work from Huard’s group reinforces this design logic: ex vivo culture expansion drives an accumulation of cellular senescence that degrades the MSC secretome and regenerative function, while enzymatic dissociation disrupts the stem-cell niche that governs potency—so a non-enzymatic, expansion-free process that keeps the niche intact is hypothesized to retain activity that conventional processing erodes [29]. Huard also recognized the limitations of the present state of orthobiologic products and addressed his progressive appreciation of preserving the niche, which he emphasized in his Research Impact Statement as part of the AAOS/OREF 2026 Arnold I. Caplan Award for Distinguished Research in Orthobiologics Title: Origin, Mechanism of Action(s) & Clinical Application of MSCs [57]. The sections that follow summarize the experimental program that has characterized this product and its components.

## 4. Engineering Product Quality: Bone Marrow Aspirate and the BMAX Program

The earliest goal of the Donner group was to identify minimally manipulated and immediately translatable improvements to traditional bone marrow-derived orthobiologic to enhance clinical outcomes. The initial concept was to increase the number of MSCs in the bone marrow aspirate and subsequent BMAC through a variety of techniques beyond those described by Hernigou, including intraosseous flushing of the bone marrow, which was determined not to be technically feasible (E.J.D., unpublished data), aspiration of bone marrow blood through a bone core within the Jamshidi needle, which yielded ~1.5× more MSCs (E.J.D., unpublished data) and identifying the location of and collecting the MSCs in the RBC layer just below the buffy coat often neglected in BMAC processing systems, yielding ~5× more MSCs (E.J.D., unpublished data) while minimizing the detrimental effects of RBCs. We further studied the biological effects and therapeutic quality of anticoagulants with different mechanisms of action, demonstrating that heparin has a better bioactive profile, as well as identifying bone marrow-derived platelets to be more pro-regenerative than peripheral blood platelets and a better source for obtaining PRP to supply a primary PRP orthobiologic. These advances were followed by the realization and central premise that the regenerative potency of a bone marrow biologic is likely determined by its composition and preparation and not by the cell number alone, leading to the development of BMAX.

### 4.1. Reducing Red Blood Cells Improves the Biologic

Red blood cells are the dominant contaminant of aspirate-based products and are not inert. In controlled in vitro experiments, erythrocytes and their releasates were directly cytotoxic to marrow-derived human MSCs, reducing viable-MSC counts at hematocrit or releasate concentrations of ≥10% within three days through necrosis, apoptosis, and lipid-peroxidation-associated ferroptosis; the authors proposed maintaining product hematocrit below approximately 10% as a quality threshold [58]. Independent in vivo work supports the same principle: erythrocyte depletion of bone marrow concentrate improved its therapeutic efficacy in a rodent OA model [59]. A processing method that minimizes red-cell content is therefore expected to yield a more potent, less pro-inflammatory product. The hematocrit of BMAX itself has not yet been measured.

### 4.2. Processing Variables Such as Anticoagulant Materially Change Bioactivity

That preparation, not merely cellularity, governs bioactivity was demonstrated directly by comparing anticoagulants: relative to sodium citrate, heparin anticoagulation of bone marrow concentrate increased colony-forming-unit fibroblast frequency several-fold (1037 versus 187 colonies/mL; *p* = 0.0001) and reshaped the secretome without changing nucleated-cell number [60]. This finding reframes an ostensibly trivial handling choice as a determinant of therapeutic quality and underscores why nominally similar “BMAC” and marrow MSC products can perform very differently.

### 4.3. Marrow-Derived Platelets Carry a Distinct Pro-Regenerative Secretome

Platelets are a major effector population in marrow biologics, and the group showed that platelets from the marrow niche differ reproducibly from peripheral-blood platelets. Normalized per platelet, marrow-derived platelets secreted markedly more hepatocyte growth factor (~20-fold; day 3, 6509 ± 1130 versus 275 ± 70 fg per 10^6^ platelets; *p* < 0.001), together with elevated macrophage colony-stimulating factor, interleukin (IL)-10, IL-4, IL-1 receptor antagonist protein (IRAP), and arginase-1—a coordinated, macrophage-M2-polarizing, anti-inflammatory secretory profile [61]. Preserving and concentrating marrow-niche platelets, rather than diluting them with peripheral blood, is thus a rational design goal, with the further option of augmentation using bone marrow-derived platelet releasates. These data were obtained from isolated platelets; the platelet content and secretion of BMAX itself have not yet been measured.

### 4.4. Ex Vivo Characterization of the Micronized Product

Bringing these principles together, ex vivo characterization of BMAX showed that mechanical, non-enzymatic processing preserving the native stroma yields a product functionally distinct from centrifuged BMAC (Table 1) [62]. BMAX yielded 46,050 ± 6338 nucleated cells per mg of bone core at 89.4% viability, versus 76,188 ± 8358 cells per µL at 95.8% viability for BMAC; because the units differ, the yields are not directly comparable, and viability was significantly lower with BMAX (*p* = 0.0007) [62]. In culture, the BMAX plastic-adherent fraction reached significantly higher culture density (34.9 ± 4.2 versus 20.0 ± 1.8 × 10^3^ cells/cm^2^ at P0 confluence; *p* = 0.017), was enriched over passage for CD146^+^CD45^−^ pericyte-like cells in the single-donor study described in Section 5 (significantly exceeding matched BMAC at passage 2; *p* = 0.0446), although a three-donor pilot found no difference in pericyte-like frequency at any passage [62], and mounted a distinct secretory response to inflammatory (TNF-α) challenge, including markedly higher IL-6 (a pleiotropic IL often associated with inflammation but critically important for maintaining the bone marrow niche) and lower leukocyte chemoattractant chemokine IP-10 while retaining IRAP [62]. These secretome data describe cells after eight days of culture under TNF-α stimulation and are not concentrations in the administered product. In short, these ex vivo data indicate that BMAX trades bulk cellularity for an adherent, matrix-associated population with a distinct inflammatory-response secretome.

No quantitative protein profile of the administered product is yet available. Concentrations of growth factors, cytokines, and matrix-associated signaling proteins in the injectate as prepared—with defined analytical methods, units, normalization, and donor-to-donor variability—have not yet been measured, and the composition and functional claims made for BMAX in this Perspective should be read with that limitation in mind. Properties inferred from marrow biology rather than measured in the product are identified as such in Table 1.

### 4.5. Megakaryocytes and Hematopoietic Stem/Progenitor Cells: A Hypothesized Contribution

Because BMAX preserves marrow parenchyma rather than a purified cell fraction, it is expected to retain populations that culture expansion eliminates entirely—most notably megakaryocytes (MKs) and hematopoietic stem/progenitor cells (HSPCs), both now recognized as far more than lineage-committed blood cells. Both are also present in aspirate-based products; the distinction we propose is one of context rather than presence, since in BMAX they would be delivered still associated with their native matrix and with MSCs and pericytes rather than dissociated in suspension, which may favor the cell–cell and cell–matrix communication described in Section 2. Neither population has yet been quantified in the micronized product, and this hypothesis remains to be tested. Megakaryocytes are active regulators of the stem-cell niche rather than mere platelet factories: they lie directly adjacent to HSPCs and maintain their quiescence through CXCL4/PF4 and TGF-β1, and after marrow injury they switch to secreting FGF1 to drive HSC expansion and regeneration [63,64]. They are also bona fide immune cells—roughly a fifth of marrow MKs express MHC class II with CD80/CD86/CD40, present antigen to CD4^+^ T cells, and bias them toward a regulatory, anti-inflammatory (Treg) fate via TGF-β1, while emperipolesis, the transit of live neutrophils through the megakaryocyte cytoplasm, provides a route for bidirectional immune exchange [65,66]. A recent review in this journal accordingly frames megakaryocytes as critical integrators of hemostatic, immune, and regenerative processes with therapeutic potential for modulating inflammation [65].

Hematopoietic stem/progenitor cells likewise contribute more than blood-cell renewal. They sense infection and inflammation directly through Toll-like and cytokine receptors and mount emergency myelopoiesis, positioning the HSC as a foundation of the immune response rather than a passive precursor [67,68], and inflammatory exposure imprints a durable, epigenetic trained-immunity memory on their innate output [69]; that megakaryocytes and an HSPC reservoir co-localize even outside the marrow underscores that these lineages travel and function as a unit [70]. If BMAX retains megakaryocytes—with their HSC-supportive, Treg-inducing activity—and HSPCs—with their regenerative and immune-sensing capacity—alongside pericytes and MSCs, it would deliver an intact parenchymal unit rather than a disaggregated or monocultured suspension, a hypothesis that could help explain why the whole niche might outperform an isolated cell, and one that requires direct quantification of these populations in the product.

**Table 1:** Characterization of conventional bone marrow aspirate concentrate (BMAC) and the niche-preserving micronized preparation (BMAX), grouped by the strength of the evidence. (**A**) Parameters measured in the product as prepared. (**B**) Parameters measured only after culture or inflammatory (TNF-α) stimulation, which describe cell behavior ex vivo and are not concentrations in the administered product. (C) Properties expected from the processing design or from marrow biology that have not yet been measured in BMAX. Data in [62] are from a pilot series of three donors (secretome comparisons by one-tailed *t*-test); data in [71] are from a single donor. Where the two studies differ, both results are shown. ns, not significant.

Parameter	Conventional BMAC	BMAX (Niche-Preserving)
Processing	Aspiration + centrifugation to a buffy-coat concentrate	Mechanical, non-enzymatic micronization of marrow with trabecular bone and stroma
**A. Measured in the product as prepared**		
Total nucleated cells	76,188 ± 8358 per µL	46,050 ± 6338 per mg of bone core; units differ, so yields are not directly comparable [62]
Viability (calcein-AM)	95.8 ± 0.7%	89.4 ± 1.5% (*p* = 0.0007) [62]
**B. Measured after culture or inflammatory stimulation (not concentrations in the injectate)**		
Adherent-cell density at P0 confluence	20.0 ± 1.8 × 10^3^ cells/cm^2^	34.9 ± 4.2 × 10^3^ cells/cm^2^ (*p* = 0.017) [62]
ISCT MSC phenotype (CD73^+^CD90^+^CD105^+^), % of adherent cells	90.0 ± 3.3% at P3	86.9 ± 6.7% at P3 (ns) [62]; no difference at P0 [71]
Pericyte-like cells (CD146^+^CD45^−^), % of adherent cells	Donor-matched reference	No difference at P0–P3 in three donors [62]; increased over passage, significant at P2 (*p* = 0.0446), in one donor [71]
Colony-forming units–fibroblast (day 14)	Donor-matched reference	More colonies [71]
Secretome after TNF-α challenge (2.5 ng/mL; 13-plex bead immunoassay; pg/mL normalized per 10^6^ cells plated; day 8)	IL-6 46.5 ± 22.6; IP-10 80.6 ± 31.0	IL-6 6849 ± 3644 (*p* = 0.050); IP-10 0.72 ± 0.35 (*p* = 0.050); IRAP comparable to BMAC by day 8 [62]
**C. Expected from processing design or marrow biology; not yet measured in BMAX**		
Red blood cells	High (red-cell-rich)	Reduction is a design goal (RBCs cytotoxic to MSCs at ≥10% hematocrit in vitro [58]); product hematocrit not yet measured
Leukocytes (WBC)	High	Expected to be lower, inferred from lower nucleated-cell yield [62]; not directly counted
Platelets	Peripheral-blood-diluted	Marrow platelets secrete ~20-fold more HGF than peripheral platelets [61]; platelet content of BMAX not yet measured
Megakaryocytes	Present in aspirate; not quantified here	Expected to be retained in association with native matrix [63,64,65,66]; not yet quantified
HSPCs	Present; hematopoietic cells make up ~99% of BMAC nucleated cells [62]	Expected to be retained in association with native matrix [67,68,69,70]; not yet quantified
Extracellular matrix/niche	Disaggregated; cells stripped into suspension	GAG-rich stromal particles present after processing, from which adherent cells emerge in culture [62]; matrix composition not yet quantified
Quantitative protein profile of the injectate (growth factors, cytokines, matrix-associated proteins)	Not reported here	Not yet measured
Release specifications; donor- and batch-to-batch variability	Not systematically characterized	Not yet characterized (Section 7)

## 5. Preclinical Efficacy: BMAX Versus BMAC in Post-Traumatic Osteoarthritis

These ex vivo advantages motivated a controlled in vivo experiment, recently published in this Special Issue [71]. In a destabilization-of-the-medial-meniscus (DMM) model of PTOA in immunodeficient (RNU nude) rats—an established rodent approach for OA-pain research [72]—human donor-derived micronized bone marrow (BMAX) was compared head-to-head with patient-equivalent BMAC and with phosphate-buffered saline (PBS) vehicle (*n* = 10–12 per group), delivered by intra-articular injection four weeks after injury and assessed through eight weeks. At eight weeks, BMAX significantly improved femoral-condyle Osteoarthritis Research Society International (OARSI) histological scores relative to vehicle (*p* = 0.0020) and, importantly, relative to BMAC (*p* = 0.0243), while both cell therapies improved tibial-plateau scores; proteoglycan and type-II collagen staining were better preserved with BMAX, which also reduced movement-evoked (knee-bend) pain relative to both vehicle (*p* = 0.0027) and BMAC (*p* = 0.0090) and reduced joint swelling relative to vehicle (*p* = 0.0196) [71]. The study concluded that “preservation of the native bone marrow ECM was associated with improved functional outcomes and greater cartilage regeneration compared with conventional BMAC in this preclinical rat model of post-traumatic osteoarthritis” [71]. To our knowledge, this is the first controlled preclinical demonstration that a niche-preserving micronized marrow product outperforms conventional BMAC. It is consistent with, but does not establish, the hypothesis that loss of the niche contributes to the underperformance of conventional BMAC in clinical trials (Section 1). Because these gains were structural—improved OARSI histology with better-preserved proteoglycan and type-II collagen—rather than analgesic alone, they may represent an early disease-modifying signal in this model, although histological endpoints at eight weeks in a rodent cannot establish disease modification in patients.

Two design features bear on the interpretation of these findings. First, the human donor was deliberately selected as a worst-case source: candidate donors were prospectively screened for senescent-cell burden, and the donor used was a 50-year-old woman with Ehlers–Danlos syndrome and a high senescent-cell load—an “aged,” compromised source from which regenerative performance would be expected to be poor. That BMAX from such a donor nonetheless outperformed BMAC suggests the effect is not an artifact of an unusually favorable donor, although a single donor cannot indicate how the product performs across donors. This reading aligns with Huard’s demonstration that donor aging and senescent-cell accumulation progressively impair stem-cell regenerative capacity—and that secreted pro-angiogenic factors from young donors can restore it—in accelerated-aging models [73]; a senescence-laden, worst-case donor is therefore a deliberately stringent test of the product. Second, the use of a xenogeneic human product in an immunodeficient host isolates the biologic’s intrinsic activity from adaptive-immune confounding, at the cost of removing the immune interactions that will operate in autologous clinical use. The translational relevance of the model is supported by evidence that rat and human red marrow are structurally comparable [74].

Several constraints limit the conclusions this study can support. Because BMAX differs from BMAC simultaneously in cellular composition, leukocyte and red-cell content, platelet populations, secretome, and tissue architecture, the design cannot attribute the observed benefit to niche preservation specifically; any of these differences, alone or in combination, may contribute. The study used a single human donor, a modest sample size (*n* = 10–12 per group), an immunodeficient xenogeneic host, a single dose without dose–response assessment, and follow-up to eight weeks after treatment, and the injectate was characterized functionally rather than by its cellular and protein content. The findings therefore justify further testing; they do not demonstrate that niche preservation explains the disappointing clinical results of conventional orthobiologics.

## 6. Broader Regenerative Potential

The applications discussed in this section are research opportunities rather than anticipated clinical indications; with the exception of articular cartilage, none has been tested with BMAX. Multiple articles have emphasized the regenerative potential of MSCs for a myriad of medical conditions, most notably a recent 2025 comprehensive review article, MSCs in Treating Human Diseases: Molecular Mechanisms and Clinical Studies, in which a diverse range of diseases were thoroughly reviewed with a conclusion supporting MSCs “potential for transforming the field of regenerative medicine as well as immune-mediated inflammatory diseases” [75]. Although the evidence remains hypothesis-generating, BMAX’s preservation of intact marrow parenchyma, rather than isolated cells, provides a rationale for investigating it in conditions marked by a failed transition from inflammation to repair (Table 2). Tendons, ligaments, and muscles share similar failed-healing biology, and adult regenerative cells supporting these tissues are of perivascular origin and act largely through paracrine, pro-angiogenic signaling rather than direct engraftment [55,56,76]. Huard’s group established this mechanism directly—identifying pericytes and myoendothelial cells within the walls of human blood vessels as a source of highly regenerative MSC-like cells [76], and showing that transplanted stem cells drive repair chiefly by recruiting host cells through neo-angiogenesis, with only a small fraction of donor cells engrafting and inhibition of angiogenesis abolishing the benefit [73,76]—the empirical foundation for Caplan’s “Medicinal Signaling Cell” concept [56]. The evidence is strongest for intra-articular cartilage repair, where controlled preclinical data exist [71], and progressively more speculative for the extra-articular routes summarized in Table 2; the intervertebral disk is the most developed of these, with an active clinical-trial landscape [77,78,79,80], and the broader relevance of stem-cell niches across tissues is considered elsewhere in this Special Issue [23,81], including a companion review of the immune- and lymphangiogenic-privileged limbal (corneal) stem-cell niche [82].

Moreover, the reach of a niche-preserving marrow product need not be confined to the site of injection. Delivered MSCs are not static: they home to sites of injury and inflammation along chemokine gradients [83], and beyond local differentiation they act through several non-local mechanisms increasingly recognized as central to their benefit—fusing with resident host cells to form functional heterokaryons that can reprogram and rescue damaged tissue [84]; donating healthy mitochondria to stressed recipient cells through tunneling nanotubes and vesicles, restoring cellular bioenergetics in a manner of growing interest for degenerative and age-related disease [85,86]; exerting durable effects at a distance through a paracrine secretome and extracellular vesicles [87]; and reprogramming the local and systemic immune milieu toward repair, notably by inducing regulatory T cells and shifting macrophages toward an anti-inflammatory phenotype [88]. Preserved marrow B-lineage populations may add a further immunoregulatory dimension: regulatory B cells (Bregs) are a heterogeneous, context-dependent subset that restrain inflammation through anti-inflammatory cytokines, inhibitory surface molecules, and interactions with T cells and dendritic cells, and their dysfunction can act as both a driver and a consequence of autoimmune pathology in a disease-stage- and microenvironment-dependent manner [89]. Whether a niche-intact product such as BMAX engages these migratory, fusogenic, mitochondrial-transfer, paracrine, and immunomodulatory routes has not been examined; each remains hypothesis-generating in the specific context of BMAX and defines a mechanistic research agenda rather than an established clinical effect.

Because this regenerative activity is substantially paracrine, the same niche-preserving product also opens an acellular route: the BMAX secretome, including extracellular vesicles (EVs; exosomes), could deliver its pro-regenerative, anti-inflammatory cargo without live cells; in osteoarthritis, MSC-derived EVs have been shown both to protect cartilage and bone from degradation and to promote human cartilage matrix regeneration—suppressing chondrocyte inflammation while stimulating proteoglycan and type-II-collagen synthesis—in preclinical models [90,91]. A BMAX-derived exosome preparation could, in principle, offer an off-the-shelf format: storable, dose-standardized, and manufacturable at scale, and—whether autologous or allogeneic—would decouple the treatment from an individual patient’s donor quality, thereby sidestepping the very donor-age and senescence limitations that erode autologous cell products—a prospect worth investigating given the rising prevalence of older and metabolically compromised donors who most need treatment. This acellular direction remains hypothesis-generating and will require dedicated characterization and controlled testing, but it follows directly from the same principle that motivates BMAX: preserve and deploy the regenerative signaling unit rather than an isolated cell.

Bone healing is a further research opportunity: autologous bone-marrow concentrate already has an established clinical track record in accelerating fracture repair and resolving nonunion, where outcomes scale with the number and concentration of marrow progenitor cells delivered [92]. Because BMAX is designed to retain the matrix-embedded, osteoprogenitor-rich stromal fraction that aspiration dilutes—and because its exosome derivative would carry the same natively osteogenic, pro-angiogenic signaling—both the cellular product and an EV preparation could be investigated for fracture and nonunion repair, complementing their intra-articular application. This niche-preserving approach also lends itself to banking: recent work from Huard’s group shows that short-term cryopreservation does not compromise the function of MSCs from bone marrow concentrate for cartilage repair, raising the possibility of a single BMAX harvest that can be stored and divided across multiple injections [93].

## 7. Limitations and Translational Considerations

Several limitations qualify the interpretation offered here. First, the comparative efficacy evidence for BMAX is preclinical and rests on a single controlled study in a rodent post-traumatic OA model [71]; animal models frequently fail to predict human outcomes, and the demonstrated superiority over BMAC, while encouraging, cannot by itself support claims of clinical superiority or of disease modification in patients. The biological importance of the marrow niche—its ECM, perivascular populations, megakaryocytes, HSPCs, and paracrine signaling—is well established, but demonstrating that these components matter in situ does not establish that preserving them in a micronized preparation reproduces their function after intra-articular injection. Biological plausibility is therefore a rationale for testing BMAX, not evidence of efficacy, and the same evidentiary standard applies to BMAX as to the conventional products against which it is compared.

Second, important translational gaps remain. The pivotal study used a xenogeneic human product in immunodeficient rats, a design that isolates intrinsic bioactivity but removes the adaptive-immune interactions that will operate in autologous human use; species differences in marrow architecture, joint size, dosing, and mechanical loading, together with the challenge of scaling dose from a rodent knee to a human joint, are not yet addressed. Cell and tissue retention after intra-articular delivery is a general limitation of all injectable orthobiologics and may constrain BMAX as it does other products, independent of processing. Moreover, the disappointing results of BMAC, PRP, SVF, and allogeneic MSCs may reflect not only processing but also factors intrinsic to OA biology—patient age, disease heterogeneity and severity, the inflammatory joint microenvironment, and limited cell retention—as well as trial-level factors such as patient selection, variability in dose and preparation, and differences in rehabilitation protocols—and durable symptomatic relief without structural change may itself be a clinically meaningful outcome. These alternative explanations are not resolved by the present evidence and warrant consideration alongside the niche-preservation hypothesis.

Third, practical and manufacturing questions central to any point-of-care biologic remain open for BMAX: operator-to-operator reproducibility, batch-to-batch and donor-to-donor variability, procedural complexity and scalability, release specifications and quality-control metrics (for example, cellular composition, viability, secretome consistency, and a quantitative protein profile of the injectate), and regulatory classification. Because reproducibility and standardization are themselves central to the argument that product quality drives outcome, these parameters will need to be characterized and reported, and the product’s composition and variability defined, as summarized in Table 1.

Fourth, the extra-articular and acellular applications discussed in Section 6—tendinopathy, ligament and disk disease, peripheral neuropathy, spinal cord injury, systemic inflammatory conditions, fracture repair, and extracellular-vesicle products—are hypothesis-generating extrapolations from shared biology rather than demonstrated indications, and each would require its own preclinical and clinical validation before being considered a realistic translational target.

Finally, translating this concept will require independent, externally conducted studies and appropriately designed clinical trials. Given E.J.D.’s involvement in the development of BMAX and the involvement of both authors in several of the cited studies, independent replication is particularly important. Future trials should specify patient selection and OA severity, dosing and injection regimen, and an active comparator—ideally head-to-head against conventional BMAC as well as corticosteroid—and should report both symptomatic endpoints and objective disease-modification measures (quantitative or compositional MRI, structural, and biomarker outcomes) over follow-up long enough to detect structural change. Only such data can establish whether the preclinical advantage reported here translates into clinical benefit.

## 8. Conclusions and Perspectives

The regenerative process is complex, tightly regulated and dynamic, requiring a highly organized network to enable effective multicellular communication and re-establish homeostasis. Regenerative medicine has become a highly studied and more accepted potential therapy for a variety of medical maladies, mostly for musculoskeletal (MSK) diseases including OA. The failure to obtain FDA approval for regenerative medicine biologics with one exception, as well as the failure of conventional orthobiologic injectables to demonstrate reliable clinical benefit in OA across multiple systematic reviews, to outperform corticosteroid in knee OA—including high-dose allogeneic umbilical-cord products—or to demonstrate clinically significant disease-modifying effects in the recent level 1 MILES study, need not be read as a refutation of regenerative medicine; among several plausible explanations (Section 7), we propose that one contributor is the delivery of a product lacking key regenerative cell populations in their niche and the ECM through which they communicate. The common form of BMAC is low in MSCs and also contains pro-inflammatory RBCs and WBCs, which may have a deleterious effect on pain, swelling and cartilage regeneration. The BMAX program is designed to invert that approach—to reduce red and white cells while retaining the marrow-platelet secretome, the ECM, and the matrix-embedded stromal populations that aspiration recovers poorly—although several of these properties have yet to be measured in the product. In a single controlled preclinical study using one deliberately compromised donor, both BMAX and BMAC significantly outperformed the vehicle PBS and, more interestingly, BMAX significantly outperformed conventional BMAC. These findings do not establish clinical efficacy, reproducibility across donors, or superiority in patients; they do identify a specific, testable approach to building a bone marrow biologic and help define the confirmatory preclinical and clinical program required to test it in OA. Applications beyond OA, including other musculoskeletal and inflammatory conditions and extracellular-vesicle products, remain research hypotheses. The regulatory classification of BMAX, which depends on factors beyond processing method alone, has not been determined. If confirmed, this approach would suggest that the next advance in the field may come less from a novel or induced cell than from the disciplined preservation of the regenerative marrow unit evolution has already assembled.

## Figures and Tables

**Table 2:** Rational applications of a niche-preserving marrow orthobiologic. Rows below articular cartilage are hypothesis-generating and define a research agenda rather than asserting established efficacy.

Target	Rationale	Delivery	Evidence State
Articular cartilage/PTOA/osteochondral defects	Preserved perivascular niche and ECM signaling; superiority to BMAC in vivo	Intra-articular; intra-osseous; pelleted into osteochondral defects	Controlled preclinical (this Special Issue) [71]
Tendinopathy/ligament	Shared failed-healing pathophysiology; hypovascular target	Peritendinous/intralesional	Hypothesis-generating
Discogenic pain	Avascular, immune-privileged, nutrient-limited niche	Intradiscal; annular	Hypothesis-generating; active RCT landscape [77]
Radiculopathy/peripheral neuropathy	Perineural inflammation; EV-mediated support	Perineural/DRG/intravenous	Hypothesis-generating
Spinal cord injury	Paracrine neuroprotection; scar modulation	Epidural; intrathecal	Hypothesis-generating
Systemic inflammatory disease	Immunomodulation via regulatory-T-cell and EV populations	Intravenous	Hypothesis-generating
Wounds	Trophic factors	Injected/Topical	Hypothesis-generating

## Data Availability

No new data were created or analyzed in this study. Data sharing is not applicable to this article.
